# Analysis of Taiwan’s Mask Collection and Plan Evasion during the COVID-19 Pandemic

**DOI:** 10.3390/ijerph18084137

**Published:** 2021-04-14

**Authors:** Po-Sheng Ko, Jen-Yao Lee

**Affiliations:** 1 Department of Public Finance and Taxation, National Kaohsiung University of Science and Technology, Kaohsiung 80778, Taiwan; psko@nkust.edu.tw; 2 Department of International Business, National Kaohsiung University of Science and Technology, Kaohsiung 80778, Taiwan

**Keywords:** mask shortage, planned quota, plan evasion

## Abstract

This study established a two-stage dynamic game strategy to analyze how the planned quota and price of masks were set and why mask manufacturing firms on the national mask team (NMT) in Taiwan evaded the plan. Plan evasion occurred when the NMT decided to produce less than the quota set by the government, even though they were incentivized and able to produce more. Taiwan’s experience shows that through the collection of masks and the Name-Based Mask Rationing System, the people’s right to procure masks can be guaranteed; however, to promote market transaction efficiency, the government should adopt a lower quota for the collection of masks and allow firms to freely sell them in the market after they complete their plans. The self-interest of the government played a key role in inducing plan evasion.

## 1. Introduction

At the beginning of 2020, the COVID-19 pandemic had already reached a global scale. The primary route of transmission of COVID-19 is via small respiratory droplets, and presymptomatic and asymptomatic individuals are able to transmit the virus to others. Karaivanov et al. [1] estimated the impact of mandatory mask wearing and other non-pharmaceutical interventions on the growth of COVID-19 cases in Canada by using counterfactual policy simulations. The results suggested that mandating the use of masks indoors nationwide in early July 2020 could have reduced the weekly number of new cases in Canada by 25–40 percent by mid-August, which translates into 700–1100 fewer cases per week. Mitze et al. [2] found that after face masks were made mandatory, the number of newly registered severe acute respiratory syndrome coronavirus 2 infections decreased by between 15% and 75% over a period of 20 days. The authors concluded that face masks reduced the daily growth rate of reported infections by around 47%.

The unexpected and sudden pandemic led to the stockpiling and panic-buying of masks by people in Taiwan, causing a face mask shortage; thus, the shortage of masks, soaring prices, and equity of allocation were important issues in the early phase of the COVID-19 pandemic [3]. The government took action to control the supply and allocation of face masks, and this unique mask plan was believed to have greatly contributed to the success of curbing the spread of COVID-19 in Taiwan [4,5,6]. Several strategies implemented by the government led to a relatively controllable situation in Taiwan [4,7]. Taiwan’s success in combating COVID-19 captured our attention, and the use of masks in public spaces is believed to have played a crucial role in curbing infections [5,6,8].

Since January 2020, as the epidemic of this severe and particularly infectious pneumonia has become more severe, confirmed cases have also appeared in Taiwan. The Taiwanese government had concerns that once China controlled the export of masks, Taiwan’s self-produced mask supply would be insufficient. After Taiwan’s medical institutions and people purchased a large number of masks to prevent and control infections that occurred through droplets, there was a shortage of masks.

Consequently, in order to ensure a stable supply of masks, the Taiwanese government announced a ban on mask exports on 24 January 2020, also announcing that it would invest more than NT$200 million to build 60 mask production lines to expand its production capacity. In addition, it announced the requisition of masks and implemented the Name-Based Mask Rationing System on February 6. When the requisition of masks was announced, the total production capacity of Taiwan’s mask manufacturers was 1.88 million pieces in 11 h of operation and 4 million pieces within 24 h. The 60 mask production lines set up by the Taiwanese government were under the guidance of the private sector, with each production line capacity based on a daily production of 100,000 masks; thus, the daily production capacity could be increased by 6 million to a total of 10 million per day. These firms were collectively called the National Mask Team (NMT) [9].

After that, on 1 June 2020, as the Taiwanese government confirmed the volume of face masks that it needed to requisition, it finalized its plan to open up mask exports and allowed manufacturers to sell any masks produced beyond the number requisitioned by the government to domestic and overseas buyers [10].

However, a number of mask manufacturing firms in the NMT were found to have privately set up mask production lines to produce and sell masks for profit during the controlled requisition period. For example, in late September 2020, Deer Masks Technology Co. Ltd. was found to have set up unlicensed mask production equipment to produce masks [11].

Although the planned collection allowed the public to buy masks at a lower price, the difference between the official price and the market price also led to the phenomenon of plan evasion by the NMT. An important issue is whether plan evasion ought to exist and why it occurred. A benevolent government planning to expropriate such supplies can benefit the people, but a self-interested government may create a rent-seeking scenario, especially given the shortage of medical supplies during the COVID-19 pandemic period.

In this paper, we provide a game analysis to explore why plan evasion might occur when the NMT decides to produce less than the quota established by the government, even though they are incentivized and able to produce more. The self-interest of the government plays a key role in the plan evasion.

The organization of the paper is as follows. The literature review provided in Section 2 and Section 3 discusses the output decision of the NMT under perfect information. Section 4 further discusses the issue of plan evasion under imperfect information, while Section 5 provides conclusions.

## 2. Literature Review

The COVID-19 pandemic caused a global shortage of medical masks, leaving many health personnel exposed without appropriate protection. The World Health Organization (WHO) warned of possible shortages of face masks due to rising demand, panic buying, hoarding, and misuse [12]. The government needed to provide clear and consistent criteria to ensure the widest availability and appropriate use of facial protection, bearing in mind the challenges faced by populations in socio-economically disadvantaged settings [13]. Policymakers needed guidance on how masks should be used by the general population to combat the COVID-19 pandemic [14]. Some researchers believed that the required masks could be produced through 3D printing [15,16,17]. Ma et al. [18] suggested that reusing these masks could minimize waste, protect the environment, and help solve the imminent shortage of masks; however, allocating the use of medical resources through a reasonable economic system was also an important issue.

The WHO also recommended strategies to optimize the availability of personal protective equipment [19]. Kirubarajan et al. [20] revealed numerous strategies that had been evaluated for overcoming a limited supply of personal protective equipment during pandemics or epidemics. They grouped the strategies into six main categories: (1) decontamination of disposable masks [21,22,23], (2) reuse and/or extended wear of disposable masks [24,25,26,27], (3) layering of masks [28,29], (4) introduction of reusable respirators [29,30], (5) use of non-traditional replacements or modifications to masks [31,32], and (6) use of stockpiled or expired masks [33,34,35]. However, none of the above-mentioned studies discussed the mechanism of allocating masks.

In responding to the COVID-19 pandemic, countries have faced various choices concerning the shortage of masks. One country might intervene in the mask market with a price-ceiling policy only, while another country might take full control of production and distribution of masks. The governments of the world have shared no specific policy on the production and distribution of masks, and there are no related articles available in the public health database.

An example of these disparate approaches is the Korean government’s implementation of three Emergency Stabilization Policies, one after another within a month, to stabilize the mask market [36]. The first Emergency Stabilization Policy only obliged manufacturers and sellers to report the price, volume of production, and sales to the government. The second and third Emergency Stabilization Policies obliged all mask manufacturers to supply 50% and 50–80% of their daily production to public sales outlets; in effect, the government imposed a quota of publicly available masks on these manufacturers. People were guaranteed the ability to purchase two masks per week at a price of 1500 KRW (1.25 USD), a price uniformly applied to all public sales outlets [36].

In Taiwan, all masks were expropriated by the government, and a Name-Based Mask Rationing System was employed in which people could buy nine masks every fortnight if they were verified via their National Health Insurance card. Moreover, this mask plan safeguarded the purchase of masks and resulted in decreased anxiety in the public over a mask shortage [3].

In order to ensure a stable supply of masks, the Taiwanese government announced a ban on mask exports and invested more than NT$200 million to build 60 mask production lines to expand the production capacity of masks. Since 1 June 2020, the government has shifted from full requisition to fixed-amount requisition of masks, with a total of 8 million pieces per day, based on the production capacity allocation of each firm during the requisition period. The bans on exports and domestic retail sales of masks were lifted. After supplying the requisition quota, firms were free to sell masks domestically and overseas. Tsai et al. [3] found that the unique name-based mask rationing plan allowed for control of the production and supply of masks and contributed to their appropriate allocation. The mask rationing plan not only provided the public with physical protection, but also resulted in reduced public anxiety over mask shortages during the pandemic.

The mask production line of Taiwan’s NMT and the expropriation of private production capacity were critical means of ensuring a stable supply of masks during the COVID-19 period in Taiwan. At the same time, allowing firms to freely sell masks in the market after they had reached the planned quotas reflects the spirit of the gradual dual-track system, in which the command economy and the market economy coexist; this occurred during the economic reform period in mainland China.

However, traditionally, liberal economists believe that market shortages should be solved through price mechanisms. Some scholars believe that regulating the market by planning quotas may be a tool of government policies based on self-reliance. In their research on issues related to the setting of planned quotas, Shleifer and Vishny [37] suggested that a planner, at his or her own free will, could set prices and production quotas far below the market clearing level in order to create a shortage. In this way, the planner can capture rent through rationing. Zhou [38] also pointed out that the planner has an incentive to create shortages in the process so as to maximize rent. They will accordingly plan prices and quantities based on the optimum choice. Huang and Lee [39], expanding on Zhou’s model [38], indicated that the optimum choice of the planner would be similar to the profit maximization solution of a monopoly, where the planner pays little attention to the public interest. In the meantime, the selling price will be increased to the market-clearing price to ensure the adequacy of supply.

As a well-known step in the transition from a planned economy to a market economy, a dual-track system is usually adopted. In China’s gradual reform, economic agents include farmers, enterprises, and government authorities. Once the economic units reach the planned quota, they earn the right to sell quantities in excess of the quota and even have some freedom in setting the price. According to Koo and Obst [40], the dual-track system is the best approach during the transition in the initial stage of reform when the rule of the market has not yet been established and state-owned enterprises are regarded as monopolist. In the initial stage of economic reform, the State-Owned Enterprise is monopolistic, because competition is lacking in private sectors [39]; thus, the existence of a planned quota can help reduce the monopoly power of state-owned enterprises and ease the operation of the entire economy.

However, under the dual-track system, severe plan evasion problems have arisen in state-owned enterprises of the former Soviet Union, Eastern Europe, and China. Plan evasion occurs when state-owned enterprises, faced with production quotas regulated by the government, decide to produce output levels that are lower than the planned quota despite the fact that excess production capacity exists. Jing [41] indicated that in some situations, the phenomenon of plan evasion is rather common; for example, if the cost of an item with lower output appears to be lower, the company will choose an output level that is lower than the planned quota. Sicular [42] discussed a general equilibrium model of two economic units (the urban and the rural) and three products and demonstrated that under the dual system, decisions involving output and consumption at equilibrium depended on relative market prices, which are irrelevant to the planned quota and prices.

Tsang and Cheng [43], expanding on Jing’s model [41], found that if the individual firm is a price taker, the output will be influenced by both the market and planned prices. As such, it leads to output instability. In other words, changes in the market price and planned price cause fluctuations in the firm’s profit-maximizing output. As a result, a kinked supply curve prevails in the presence of plan evasion.

There are, however, unexplained issues in Tsang and Cheng’s paper [43]. First, in general, state-owned enterprises guarantee the production of the planned quota, that is, the state-owned enterprises’ revenue, allocated by the government for fulfilling the production plan, should cover the total cost of production, but this important issue is ignored in their model. Second, plan evasion and output instability are direct results of the fickleness of the plan [43], that is, the irrationality of the administration is the culprit behind plan evasion and output instability. In short, their model needs to be generalized to include more relevant behavioral assumptions.

In order to discuss plan evasion by the NMT, this study established a two-stage dynamic game strategy to analyze how the planned quota and price are set and why the NMT evaded the plan. In the first stage, planners decide on the purchase price and quantity; in the second stage, firms on the NMT determine the production quantity. The concept of rent seeking by government authorities, as suggested by Sheifer and Vishny [37], and State-Owned Enterprise output models by Zhou [38] and Huang and Lee [39] are both incorporated into the analysis in this paper.

In the game strategy of mask control production, firms on the NMT are like state-owned enterprises under the planned economy of the past. They must reach the planned quota before they can sell the remaining products to the market. This paper discusses plan evasion by the firms on the National Mask Team (NMT) when the government has perfect and imperfect information. We assume that NMT members are price takers and that the planner is rational (self-interest). The objectives are to (i) explore whether rational NMT members will implement plan evasion when the government has complete knowledge of their cost structure and (ii) examine the evasion issue when the government does not possess complete knowledge of their cost structure (imperfect information). Backward induction is used under the premise of Subgame Perfect Nash equilibrium.

## 3. The NMT’s Output and the Plan under Perfect Information

As mentioned in the previous section, this paper expands on both rent-seeking behavior by government authorities [37] and the state-owned enterprise’s output [38,39]. We first explore the NMT’s output decision under perfect information before analyzing the issue of plan evasion.

### 3.1. Assumptions of the Model

We use a simple model to explore the market equilibrium that allows the NMT’s excess production to be sold in the market after the planned quota is met. Due to the assumption that mask production of all the firms on the NMT is regulated by the planner, firms on the NMT are like state-owned enterprises, and every firm acts as a price-taker as well. Thus, a single firm’s choices are representative of the firms on the NMT. The two-stage sequential game strategy can therefore be modeled by a representative firm, the NMT, that has the same production behavior, and produces the same output as the entire industry.

First, firms on the NMT can sell their excess output (production beyond the quota) at market price, i.e., act as price takers. Second, the planned price $P_{0}^{}$ and planned quota $q_{0}^{}$ are determined by a planner, and NMT members are required to meet the quota and receive the total revenue $P_{0}^{}q_{0}^{}$. Third, without loss of generality, the cost function is approximated as $C\left(q\right)\approx cq_{}^{2}/2$ at a given output level via Taylor’s expansion (complicated cost functions can be linearized, and a quadratic approximation in general would suffice), and furthermore, production costs of NMT members are reimbursed from revenue, or $P_{0}^{}q_{0}^{}\geq C\left(q_{0}^{}\right)$, although for simplicity, we assume that $P_{0}^{}q_{0}^{}=C\left(q_{0}^{}\right)$, a break-even point (a constant mark-up on cost does not even alter the result qualitatively). Fourth, an NMT member can choose an excess output level $q_{s}^{}$ that it will sell in the free market after meeting the quota requirement, so the total output of the NMT is $q_{g}^{}=q_{0}^{}+q_{s}^{}$. Fifth, the planner is supposed to distribute output equally to consumers at $P_{0}^{}$, but in actuality, the goods are often rationed to those who pay bribes or are of special interest to the planner. Zhou [38] indicates that the utility function of the local government is an increasing function of the economic rent; however, we assume that the utility function is an increasing function of the difference between the market price and planned price. Thus, the size of the bribe or other economic gains can be expressed as $\left[P_{m}^{}–P_{0}^{}\right]q_{0}^{}$, where $P_{m}^{}$ is the market price. The planner then distributes the output to consumers. If the demand at the planned price exceeds the planned output, rationing is necessary. The self-interested planner can extract bribes, inducement payments, or political capital from consumers in the rationing process. Finally, aside from the planner’s personal gains, the public interest cannot be ignored; the degree to which the public interest is heeded is denoted by α (0≤α≤1). It should be pointed out that consumers buy a product at the price $P_{m}^{}$ regardless of where they buy it. Consequently, consumer surplus is fixed and, as such, is not considered in the analysis.

Given these assumptions, the output decision of NMT members can be considered a two-stage sequential game strategy. Initially, by taking into consideration the excess output, the planner will choose a price and output in order to maximize the objective function. Next, NMT members may choose the output level (sum of the planned output and excess output) that maximizes their own profits. As an alternative, NMT members may opt for planned evasion; that is, the profit-maximizing solution is achieved based on the planned price, and the choice depends on which decision makes more profit. We also assume that the planner has perfect information about the NMT members and thus considers their reaction function (relationship between planned quota and excess output) in the process of profit maximization. This is equivalent to finding the subgame equilibrium under perfect information. A solution can be obtained via backward induction.

### 3.2. The NMT’s Output Decision

There are two scenarios in which the two-stage sequential game strategy is set. In scenario I, the NMT is expected to fulfill the planned output level. In contrast, the NMT will partially fulfill the plan (plan evasion) in scenario II. We present the formulation in the following cases.

**Case** **1.** *Optimum excess output when the NMT has achieved the planned quota.*

Let $q_{0}^{}$, $q_{s}^{}$, $P_{m}^{}$, and $\pi_{}^{}$ be planned output, excess output, market price, and profit from the excess output, respectively. Hence, the revenue from the excess output is $P_{m}^{}q_{s}^{}$, and the revenue from the plan is $P_{0}^{}q_{0}^{}$. In addition, the total output of the NMT $q_{g}^{}$ is the sum of the planned and excess outputs, or $q_{g}^{}=q_{0}^{}+q_{s}^{}$. The objective function of the NMT is as follows:

(1) $$\underset{q_{s}^{}}{max.}\pi =P_{0}^{}q_{0}^{}+P_{m}^{}q_{s}^{}-c{\left(q_{0}^{}+q_{s}^{}\right)}_{}^{2}/2\;s.t.\;\;P_{0}^{}q_{0}^{}=cq_{0}^{2}/2$$

The first-order and second-order conditions are

(2) $$\frac{\partial \pi }{\partial q_{s}^{}}=P_{m}^{}-c\left(q_{0}^{}+q_{s}^{*}\right)=0$$

(3) $$\frac{\partial^{2}\pi }{\partial q_{s}^{}}=-c<0$$

From Equation (2), we can derive the response function of the NMT:

(4) $$R\left(q_{0}^{};P_{m}^{}\right)=q_{s}^{*}=\frac{P_{m}^{}}{c}-q_{0}^{}$$

**Case** **2.** *The optimum output when the NMT partially fulfills the planned quota.*

As previously stated, when the NMT practices plan evasion, it pays to choose the optimum output ${\tilde{q}}_{g}^{}<q_{0}^{}$ ($q_{0}^{}$ is the planned output) in order to maximize profit ($\tilde{\pi }$). That is, a maximization problem can be formulated:

(5) $$\underset{{\tilde{q}}_{g}^{}}{max.}\tilde{\pi }=P_{0}^{}{\tilde{q}}_{g}^{}-c{\tilde{q}}_{g}^{2}/2\;s.t.\;\;P_{0}^{}q_{0}^{}=cq_{0}^{2}/2$$

which yields the conventional first-order and second-order conditions

(6) $$\frac{\partial \tilde{\pi }}{\partial {\tilde{q}}_{g}^{}}=cq_{0}^{}/2-c{\tilde{q}}_{g}^{*}=0$$

(7) $$\frac{\partial^{2}\tilde{\pi }}{\partial {\tilde{q}}_{g}^{2}}=-c<0$$

Likewise, from (6), we can derive the response function of the NMT:

(8) $$\tilde{R}\left({\tilde{q}}_{0}^{}\right)={\tilde{q}}_{g}^{*}=\frac{q_{0}^{}}{2}$$

### 3.3. The Planner’s Decisions for the Planned Output and Price

**Case** **3.** *The optimum planned output and price of the planner when the NMT has achieved the planned quota.*

To ensure that the NMT can fulfill the planned quota, the planned output should comply with the incentive compatibility constraint; that is, $\pi \geq \tilde{\pi }$. Furthermore, the planner ought to include Equations (4) and (8) as constraints in solving for the optimum planned price and output. Given this, the planner’s profit maximization problem is

(9) $$\underset{P_{0}^{},q_{0}^{}}{max.}G=\left[P_{m}^{}-P_{0}^{}\right]q_{0}^{}+\alpha \pi$$

(10) $$s.t.\;\;P_{0}^{}q_{0}^{}=cq_{0}^{2}/2\;;\pi \geq \tilde{\pi }$$

(11) $$q_{s}^{}=R\left(q_{0}^{};P_{m}^{}\right)\;;\;{\tilde{q}}_{g}^{}=\tilde{R}\left({\tilde{q}}_{0}^{}\right)$$

By invoking the Kuhn–Tucker condition, the optimum planned price and output are

(12) $$q_{0}^{*}=\frac{2P_{m}^{}}{3c}$$

(13) $$P_{0}^{*}=\frac{P_{m}^{}}{3c}$$

The profit of the NMT and the target of the planner can be obtained:

(14) $$\pi_{}^{*}=\frac{P_{m}^{2}}{18c}$$

(15) $$G_{}^{*}=\frac{\left(8+\alpha \right)P_{m}^{2}}{18c}$$

**Figure 1:**
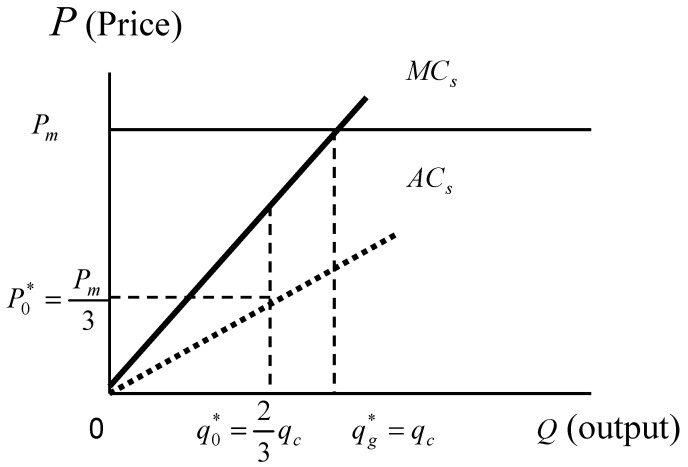
The planned output and the planned price set by the planner when the NMT fulfills the planned quota.

We can now use Figure 1 to explain Equation (12). Let $MC_{s}$ and $AC_{s}$ denote the marginal cost and average cost curves of the NMT, respectively. The planner expects that the NMT can meet the planned output and produce excess output; thus, motivated by self-interest, the planner tends to opt for a higher planned output. However, given that profit from the excess output of the NMT cannot be lower than that under plan evasion, the optimum planned output is two-thirds of the output of the competitive solution ($q_{c}^{}=P_{m}^{}/c$); that is, $q_{0}^{*}=2q_{c}^{}/3$. In other words, if the planned output is more than this output level, the NMT’s profit from the excess output will be lower than that under plan evasion. This will definitely encourage the NMT to practice plan evasion. From Equation (4), the excess output of the NMT is found to be one-third of the output of perfect competitive firms, that is, $q_{s}^{*}=q_{c}^{}/3$. It is clear that given perfect information and incentive compatibility, the NMT will produce not only the planned output, but also excess output. In other words, there will be no plan evasion, and the total output is $q_{c}^{}$. Notice that the planned output and price of this model are not affected by α.

Accordingly, we have the following lemma.

**Lemma** **1.** *The planned output and price are independent of the public interest if the planner anticipates that the NMT meets the plan.*

In the theoretical framework of this article, the objective function of the central planner includes his or her self-interest and the profit of the NMT. The self-interest component is mainly the rent obtained by the difference between the planned price and the market price; this component may be due to the political capital generated by the power to distribute masks. On the other hand, the central planner must also take into account the profits obtained by the NMT. The main reason is that the profits of the NMT will eventually enter the private economy as wages, interest, or profits.

When the planner expects that the NMT will complete the plan as scheduled, the planned price and planned output are independent of the degree of public interest, mainly because the output of the NMT will be determined by the condition of marginal revenue being equal to the marginal cost. In a competitive market, due to the constant marginal revenue, there must be a level of output at which the price (marginal revenue) equals marginal cost. At this time, the planned price and output set by the planner just maximize its rent and are independent of the degree of public interest.

**Case** **4.** *The Optimum Planned Output and Price Set by the Planner When Plan Evasion Occurs.*

To guarantee the output level when plan evasion occurs and to assure that the plan meets the incentive compatibility condition of the NMT ($\tilde{\pi }\geq \pi$), the planner needs to take Equations (4) and (8) into consideration in order to solve the optimum planned price and output. The maximization problem is

(16) $$\underset{{\tilde{P}}_{0}^{},{\tilde{q}}_{0}^{}}{max.}\tilde{G}=\left[P_{m}^{}-{\tilde{P}}_{0}^{}\right]{\tilde{q}}_{g}^{}+\alpha \tilde{\pi }$$

(17) $$s.t.\;\;{\tilde{P}}_{0}^{}{\tilde{q}}_{0}^{}=c{\tilde{q}}_{0}^{2}/2\;;\tilde{\pi }\geq \pi$$

(18) $$q_{s}^{}=R\left({\tilde{q}}_{0}^{};P_{m}^{}\right)\;;\;{\tilde{q}}_{s}^{}=\tilde{R}\left({\tilde{q}}_{0}^{}\right)$$

Using the Kuhn–Tucker condition to solve the problem, we have the following optimum planned output and price:

(19) $${\tilde{q}}_{0}^{*}=\frac{P_{m}^{}}{\left(1-\alpha /2\right)c}$$

(20) $$P_{0}^{*}=\frac{P_{m}^{}}{2\left(1-\alpha /2\right)}$$

The profit of the NMT and the target function of the planner can now readily be obtained:

(21) $${\tilde{\pi }}_{}^{*}=\frac{P_{m}^{2}}{8c{\left(1-\alpha /2\right)}^{2}}$$

(22) $${\tilde{G}}_{}^{*}=\frac{\left(2-\alpha \right)P_{m}^{2}}{8c{\left(1-\alpha /2\right)}^{2}}$$

**Figure 2:**
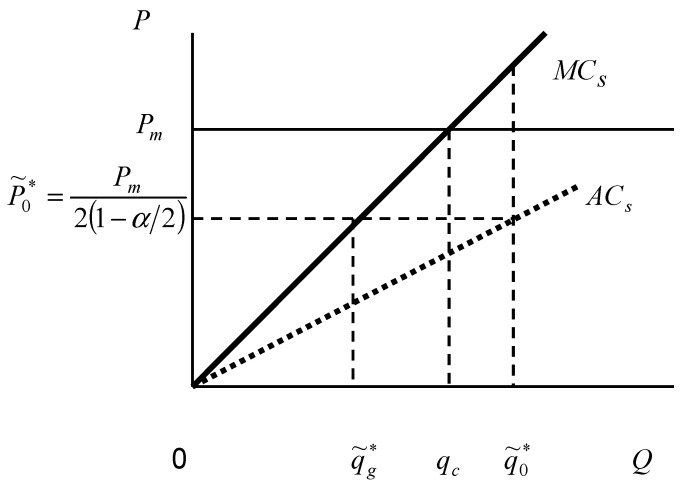
The planner’s decisions on planned quota and price under plan evasion.

We can explain Equation (19) in Figure 2 in a similar manner. When the planner anticipates plan evasion from the NMT, he or she will generally set a higher planned output (because $\frac{1}{1-\alpha /2}\geq 1>\frac{2}{3}$, so ${\tilde{q}}_{0}^{*}>q_{c}^{}$). However, the increased quota will also raise the planned price, thus reducing the marginal benefit of the quota to the planner. This prevents the planner from raising the quota without bounds. In the meantime, plan evasion by the NMT will often take place. As is evident from Equation (8), the production of the NMT is only half of the planned quota; however, there will be no production instability.

Finally, the optimum solution of the endogenous variables, including the planned output and price, correlates positively with α. Above all, from Equations (4), (8), (12) and (19), in the absence of plan evasion, the NMT’s production is that of the perfect competitive firm (that is, ${\tilde{q}}_{g}^{*}=q_{c}^{}$). On the other hand, if there is plan evasion, the total production of the NMT, given α<1, will be lower than $q_{c}^{}$ (that is, ${\tilde{q}}_{g}^{*}=\frac{1}{2-\alpha }q_{c}^{}$).

Accordingly, we have the following lemma.

**Lemma** **2.** *The planned output and price increase with the public interest if the planner anticipates that the NMT will implement plan evasion.*

When the planner expects that the NMT will evade the plan because its masks cannot be sold beyond what is stipulated in the plan, they can only be purchased by the state at the planned price. The planner will set a higher planned output and planned price to increase the profit of the NMT if the planner pays more attention to the welfare of the NMT and vice versa. When the planner ascribes equal weight to its own interests and the profit of the NMT, then its planned quota will be set at the competitive output, and when the planned price is lower than the competitive price, the NMT will inevitably respond with low-level production.

The above analysis shows that, with perfect information, the production behavior of the NMT is controlled by the planner. Given that the planner (i) designs the plan, (ii) considers the optimum response function of the NMT, (iii) complies with the incentive compatibility condition, and (iv) makes adjustments via the planned quota and price, the NMT will choose either excess production or plan evasion as the planner has anticipated.

Will the NMT opt for excess production or plan evasion? The answer is readily available: when the planner has a stronger incentive to benefit him- or herself (α<1), the payoff in Case 3 exceeds that in Case 4 ($G^{*}>{\tilde{G}}^{*}$). Thus, the planner will take into consideration both his or her and the NMT’s profit, instead of irrationally setting a high quota. The quota determined by the planner will definitely favor excess production with higher profit relative to that under plan evasion. In other words, producing excess output ensures that both the planner’s goal and the NMT’s profit are satisfied.

There is no doubt that the plan evasion described by Tsang and Cheng [43] is caused by the irrationality of the planner. In contrast, under our micro-foundation, the rational choice of the planner can preclude plan evasion by the NMT.

## 4. The NMT’s Plan Evasion under Imperfect Information

In the previous section, we discuss the issue of the planner’s plan-making and the producer’s plan evasion under complete information; however, the reality is that producers often keep information private, causing bias in the planner’s decision-making. In this section, we show that the NMT may implement plan evasion under imperfect information.

As indicated in the previous section, the optimum problem of the planner is solved with the dynamic game analysis using backward induction to achieve the subgame equilibrium in the scenario of perfect information. Nonetheless, more often than not, a planner might not fully understand the cost structure of the NMT. Generally speaking, only the NMT has full access to its own cost structure, and it may hide such information; in other words, the information on the cost structure of the NMT might well be asymmetrical between the planner and the NMT.

### 4.1. The Model

Under imperfect information, we first assume that there are two types of cost structure for the NMT. If the NMT has a high-cost structure, the cost function is expressed as $C_{}^{h}\left(q\right)=c_{h}q_{}^{2}/2$ with the probability θ. If the NMT has a low-cost structure, its cost function is expressed as $C_{}^{l}\left(q\right)=c_{l}q_{}^{2}/2$ with the probability (1−θ). Note that the type of cost structure is private information.

Second, the planner understands that there are two types of cost structure for the NMT, but is not sure whether it is a high- or low-cost structure. Given the rigidity of a planned economy and lack of selection, we assume that the planner will choose the optimum quota, which creates the greatest expected value.

Within this framework, we can apply the Harsanyi transformation and use a virtual participant (nature) for the analysis. Nature first decides the cost structure of the NMT, which the planner does not know. This imperfect information game theory translates into a game strategy with complete but imperfect information. Afterwards, we can analyze this model with the traditional method of tackling uncertainty. Given that the planner merely knows the probability distribution, he or she can only find the solution in terms of a maximum expected value. That is to say, the planner will consider the excess production of the NMT and fix a planned price and quota in order to maximize the expected value. Moreover, given the order of the plan from the planner, the NMT is expected to choose an optimum output in order to maximize the excess profit of the plan.

### 4.2. Impact of the Planned Quota on the NMT’s Excess Output Decision

As indicated in the previous section, the goal of the NMT is to maximize its profit, $\pi_{s}^{i}$, where i=h,l denotes high- and low-cost structure types. Note that for $q_{0}^{}$, which is determined by the planner, the decision variable of the NMT is the excess output $q_{s}^{i}$. Hence, the revenue from the excess output is $P_{m}^{}q_{s}^{i}$, and the revenue from the plan is $P_{0}^{}q_{0}^{}$. In addition, $q_{g}^{i}$ is the total output of the NMT, or $q_{g}^{i}\equiv q_{0}^{}+q_{s}^{i}$. Below, we discuss the problems that an NMT firm faces when it chooses to fulfill the plan or practice plan evasion.

**Case** **5.** *Optimum excess output when the NMT has achieved the planned quota.*

We start with the case in which the NMT fulfills the plan with the following objective function. Given this, the NMT’s profit maximization problem is

(23) $$\underset{q_{s}^{i}}{max.}\pi_{s}^{i}=P_{0}^{}q_{0}^{}+P_{m}^{}q_{s}^{i}-c_{i}{\left(q_{0}^{}+q_{s}^{i}\right)}_{}^{2}/2\;s.t.\;\;P_{0}^{}q_{0}^{}=cq_{0}^{2}/2$$

The first-order and second-order conditions for Equation (23) yield

(24) $$\frac{\partial \pi_{s}^{i}}{\partial q_{s}^{i}}=P_{m}^{}-c_{i}\left(q_{0}^{}+q_{s}^{i*}\right)=0$$

(25) $$\frac{\partial^{2}\pi_{s}^{i}}{\partial q_{s}^{i}}=-c_{i}<0$$

From Equation (24), we can derive the response function of the NMT:

(26) $$R_{i}\left(q_{0}^{};P_{m}^{}\right)=q_{s}^{i*}=\frac{P_{m}^{}}{c_{i}}-q_{0}^{}$$

**Case** **6.** *The optimum output when the NMT partially fulfills the planned quota.*

On the other hand, when the NMT opts for plan evasion, the objective function can be defined as

(27) $$\underset{{\tilde{q}}_{g}^{i}}{max.}{\tilde{\pi }}_{}^{i}=P_{0}^{}{\tilde{q}}_{g}^{i}-c_{i}{\left({\tilde{q}}_{g}^{i}\right)}^{2}/2\;s.t.\;P_{0}^{}q_{0}^{}=c_{i}q_{0}^{2}/2$$

Again, the first-order and second-order conditions lead to

(28) $$\frac{\partial \tilde{\pi }}{\partial {\tilde{q}}_{g}^{i}}=c_{i}q_{0}^{}/2-c_{i}{\tilde{q}}_{g}^{}=0$$

(29) $$\frac{\partial^{2}{\tilde{\pi }}^{i}}{\partial {\tilde{q}}_{g}^{i2}}=-c_{i}<0$$

Likewise, from Equation (28), we can derive the response function of the NMT:

(30) $${\tilde{R}}_{i}\left({\tilde{q}}_{0}^{}\right)={\tilde{q}}_{g}^{i*}=\frac{q_{0}^{}}{2}$$

### 4.3. Determination of the Planned Quota and Price by the Planner

When a planner designs the plan, he or she will have to take into account the following scenarios:

(1) Both firms with high- and low-cost structures fulfill the plan; (2) the firm with the low-cost structure fulfills the plan, while the firm with the high-cost structure evades the plan; (3) both types of firms evade the plan; or (4) the firm with the high-cost structure fulfills the plan, while the firm with the low-cost structure evades the plan.

Given a market price, planned quota, and planned price, the firm with a low cost will be comparatively more profitable. As such, scenario (4) is not feasible. In other words, the planner will determine the planned quota according to scenarios (1), (2), and (3). Given the types of cost structure and the corresponding incentive compatibility condition, the NMT’s choice to fulfill the plan or practice plan evasion depends on the manipulation of the central planner.

The maximum expected returns from scenarios (1), (2), and (3) for the planner and the NMT are given in Table 1. When the planner designs a plan, he or she needs to consider the incentive compatibility condition. As long as the firm with a high cost is willing to fulfill the plan, the firm with a low cost will follow suit. Thus, the incentive compatibility condition for scenario (1) becomes a constraint for the firm with the high cost. The optimum response for both firms is to produce excess output.

Similarly, if a planner hopes that the high-cost firm exercises plan evasion and the low-cost firm fulfills the plan, he or she needs to consider two incentive compatibility conditions. One is for the low-cost firm to have excess production, and the other is for the high-cost firm to opt for plan evasion.

Moreover, if the planner hopes that both firms exercise plan evasion, he or she will consider only the constraint on the low-cost firm. If the low-cost firm is reluctant to carry out the plan, the high-cost firm will definitely not fulfill it. In such a situation, the incentive compatibility condition is reduced to the constraint only on the low-cost firm.

What intrigues us most here is how the planner designs a plan. Table 1 indicates that the action taken by the planner depends on the expected return (EG).

When the payoff in scenario (1) is greater than that in scenario (2) or (3), the planner will design a plan in which both types of firms fulfill the target and have excess production. When the payoff in scenario (2) is greater than that in scenario (1) or (3), the planner will design a plan in which the high-cost firm exercises plan evasion and, at the same time, the low-cost firm fulfills the target. Likewise, when the payoff in scenario (3) is greater than that in scenario (1) or (2), the planner will design a plan in which both firms exercise plan evasion.

According to Table 1, we can find the relations among the parameters θ, α, $c_{l}$, and $c_{h}$ by comparing the three sets of expected return. First, we compare the sizes of the expected returns ($EG_{1}$) and ($EG_{2}$) for scenarios (1) and (2), respectively. It can be shown that $EG_{1}>EG_{2}$ implies

(31) $$\theta >\theta_{1}^{}\equiv \frac{\left(1-\alpha \right)\left(1-c_{l}/c_{h}\right)}{c_{l}/c_{h}\left(\alpha -0.25\right)-\left(\alpha -1\right)}$$

The reverse is true for $EG_{1}<EG_{2}$. In addition, the identical-equality sign holds for (31) in the case of $EG_{1}=EG_{2}$.

Second, for $EG_{1}>EG_{3}$, we have

(32) $$\theta >\theta_{2}^{}\equiv \frac{4\left(2-\alpha \right)-\left(16c_{l}/c_{h}\left(1-\alpha \right)+18\alpha \right)\left(1-\alpha +0.25\alpha^{2}\right)}{(1-c_{l}/c_{h})\left(-4.5\alpha^{3}+18\alpha^{2}-22\alpha +8\right)}$$

Likewise, similar conditions hold for $EG_{1}<EG_{3}$ and $EG_{1}=EG_{3}$, except that the inequality sign in (32) is replaced accordingly.

Third, in the case of $EG_{3}>EG_{2}$, we have

(33) $$\theta >\theta_{3}^{}\equiv \frac{\left(4-5\alpha +\alpha^{2}+0.25\alpha^{3}\right)}{c_{l}/c_{h}\left(2-\alpha +0.5\alpha^{2}-0.25\alpha^{3}\right)+\left(4-5\alpha +\alpha^{2}+0.25\alpha^{3}\right)}$$

Again, similar conditions hold for $EG_{3}=EG_{2}$ and $EG_{3}<EG_{2}$.

As is evident from (31), (32), and (33), the decisions of the planner are affected by the probability of knowing cost structures (θ), the planner’s concern for the public interest (α), and the difference in cost (expressed as the cost ratio $c_{l}/c_{h}$).

**Figure 3:**
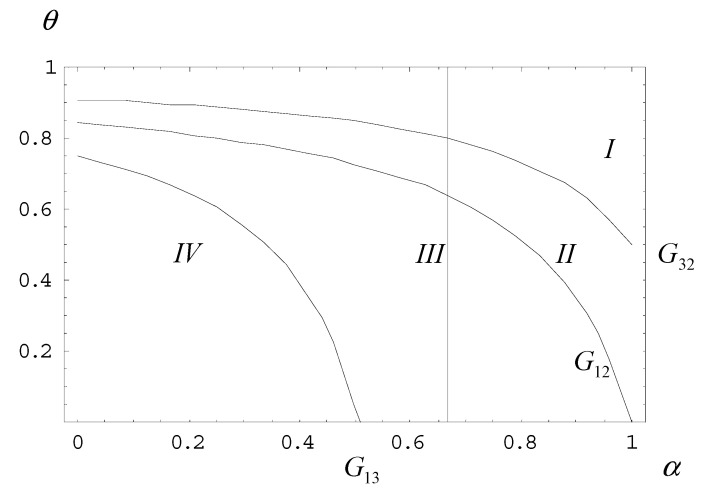
Decomposition of α, θ space for $c_{l}/c_{h}=0.2$.

Given the cost ratio, $c_{l}/c_{h}$, Figure 3 shows how the probability (θ) and the planner’s concern for the public interest (α) affect the planner’s decision for the plan.

In Figure 3, the vertical line represents the probability (θ), while the horizontal line is the planner’s concern for the public interest (α) for 0≤θ≤1 and 0≤α≤1, respectively. For $c_{l}/c_{h}=0.2$, we can sketch a graph based on Equations (31)–(33). Note that curve $G_{12}$ denotes all possible combinations of (α,θ) when $EG_{1}=EG_{2}$. Similarly, curve $G_{13}$ denotes all possible combinations of (α,θ) when $EG_{1}=EG_{3},$ and curve $G_{32}$ denotes all possible combinations of (α,θ) when $EG_{3}=EG_{2}$. As a result, the three curves partition the (α,θ) space into four sections.

In Figure 3, Region I implies $EG_{1}>EG_{3}>EG_{2}$. Given the parameter set in this area, the planner should design a plan in which both firms are expected to fulfill it in order to obtain the maximum expected return. Likewise, Region II implies $EG_{1}>EG_{2}>EG_{3}$. Similarly, in Region III, we have $EG_{2}>EG_{1}>EG_{3}$. In Region IV, we have $EG_{2}>EG_{3}>EG_{1}$ (please refer to the detailed analysis in the Appendix).

The decisions will be similar to that in Region III: Given the parameter set in this region, the planner should design a plan in which the high-cost firm practices plan evasion, while the low-cost firm fulfills the plan in order to obtain the maximum expected return. Furthermore, if the point is to the right of curve $G_{12}$ (including Region II, Region I, and curve $G_{32}$), the planner will choose a plan in which scenario (1) takes place. In contrast, if the point is to the left of curve $G_{12}$ (including Region II, Region III, and curve $G_{13}$), the planner will choose a plan in which scenario (2) will occur. The planner will not adopt any plan in which scenario (3) prevails.

Therefore, whether the NMT exercises plan evasion or excess production depends on the planner’s rational selection. When the planner becomes self-interested (α<1), they will compromise the public interest for their own in terms of adopting a high quota. This leads to plan evasion for both high- and low-cost firms. Given the cost ratio $c_{l}/c_{h}$, the volume of the planned quota determined by the planner is actually related to the probability (θ) and his or her concern for the public interest (α).

Accordingly, we have the following proposition.

**Proposition** **1.** *Both firms fulfill the plan if the probability of the NMT having a high cost is sufficiently large ($\theta >\theta_{1}$). If the probability of the NMT having a high cost is sufficiently small, the firm with a low-cost structure fulfills the plan, while the firm with a high-cost structure evades it.*

Why does the planner tend to design a plan that induces both types of firms to fulfill the plan when the parameter set (α, θ) falls on the upper-right part of curve $G_{12}$? For a given α and with high θ (the planner realizes there is higher probability of a high-cost NMT), in order to prevent plan evasion by the NMT due to the high quota, the planner tends to fix a lower planned quota to minimize his or her loss. On the other hand, for a given θ, when the planner’s concern for the public interest is high, the private interest of the planner will be largely consistent with the public interest. The planner will tend to fix a lower quota as well. This explains the reason that when the parameter set (α, θ) falls on the upper-right part of curve $G_{12}$, the planner has a tendency to design a plan that results in scenario (1), that is, to fix a lower planned quota.

In contrast, if the parameter set (α, θ) falls on the lower-left part of curve $G_{12}$, the planner is likely to design a plan that causes the low-cost firm to fulfill the plan and the high-cost firm to exercise plan evasion in order to maximize the expected return. For a given α, when the probability of a high-cost firm (θ) is low, the planner is more likely to plan a high quota, which will cause the high-cost firm to have a low expected loss. For a given θ, however, when the planner is less concerned about the public interest (α), self-interest will likely override public interest, and thus, the planner tends to opt for a plan in which scenario 2 occurs (higher planned quota).

It should be pointed out that the plan evasion described by Tsang and Cheng [43] actually results from the irrationality of the planner. In other words, plan evasion is caused by the irrationality of the authorities. In this paper, however, the analysis is based on the bedrock of a micro-foundation: rational selection. In addition, in the presence of asymmetric information on cost structures, the planner tends to impose a higher quota in order to induce the high-cost firm to evade the plan in the case of low θ and α values.

**Figure 4:**
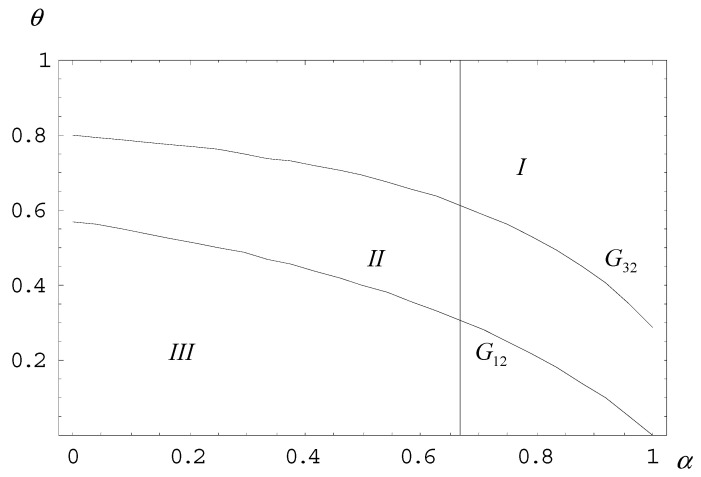
Curves $EG_{1}$, $EG_{2}$, and $EG_{3}$ (given $c_{l}/c_{h}=0.5$).

To further demonstrate how the cost ratio ($c_{l}/c_{h}$) affects the planned quota, we assume $c_{l}/c_{h}=0.5$, as shown in Figure 4. As $c_{l}/c_{h}$ approaches 1, both $G_{12}$ and $G_{32}$ (curve $G_{13}$ is not considered) gradually shift to the lower left. The result indicates that when the cost difference narrows, the planner tends to impose a plan in which scenario (1) occurs, that is, to have a lower quota in order to prevent the high-cost firm from exercising plan evasion.

Accordingly, we have the following proposition.

**Proposition** **2.** *An increasing $c_{l}/c_{h}$ and α lead to a decreasing $\theta_{1}$. Other things being equal, both firms fulfill the plan as designed by the planner if the cost ratio of the NMT and the public interest of the planner are increasing.*

An increase in $c_{l}/c_{h}$ leads to a smaller cost difference between low-cost and high-cost firms and makes the two types of firms very similar. Because the costs of both firms are much closer, for a given probability of the high-cost firm, the expected loss of the planner due to plan evasion by the high-cost firm is relatively large. The planner will choose the scenario in which both firms fulfill the plan to avoid plan evasion by the high-cost firm.

If the planner’s concern for the public interest α is increasing, a greater weight is placed on the firm’s profit, so the expected loss of the planner is determined by the increasing probability of plan evasion by the high-cost firm, so the planner will choose the scenario that avoids plan evasion by the high-cost firm.

### 4.4. Discussion

The main argument of this study is that planners have self-interested motives when determining the planned quota of masks to be collected. At the same time, the profit level of the NMT must also be taken into account. With complete information, even if the central planners are less concerned about the public interest, they will still set a level of expropriation that will not lead to plan evasion, because the public benefits due to excess production can always compensate for the loss of personal benefits.

However, when the cost information is incomplete, the planner’s best options are the following: (1) both firms with high- and low-cost structures fulfill the plan; or (2) the firm with the low-cost structure fulfills the plan, while the firm with the high-cost structure evades it.

On the one hand, when employing a plan that results in both high- and low-cost firms fulfilling it, the planner sets a lower planned quota to stimulate both high-cost and low-cost firms to meet the planned quota and incorporate any excess output. At this time, planners receive a low allocation of masks, but they can meet the needs of the public interest through the excess output of firms. On the other hand, when the planner imposes a plan that causes the low-cost firms to fulfill it while high-cost firms evade it, the planned quota is relatively high. The low-cost firms still choose to complete the planned quota and sell the excess output to the market; however, the high-cost firms will not complete the planned quota due to the high production cost of the excess output, so they will have low-level production and be unwilling to overproduce.

According to the above analysis, to minimize the expected loss and enhance welfare, a planner is more likely to impose a plan in which both firms with high- and low-cost structures fulfill the plan when (i) the probability of the high-cost firm (θ) is high; (ii) the planner is more concerned about the public interest (α); and (iii) the cost difference is narrow (the cost ratio $c_{l}/c_{h}$ is larger).

Tsai et al. [3] asserted that Taiwan’s mask rationing plan (collection of masks and the Name-Based Mask Rationing System) not only provided the public with physical protection, but also resulted in reduced public anxiety over mask shortages during the pandemic. In contrast to the views of Tsai et al. [3], this study finds that artificially created shortages may arise in the process of mask collection, especially when the planning authority is unable to acquire the cost information of mask manufacturers.

Taiwan’s successful control of the COVID-19 epidemic is less the result of the collection of masks and the Name-Based Mask Rationing System and more due to border control measures that were more successful than those of other countries.

Taiwan’s experience tells us that even with the mask collection system, there may still be a supply shortage. At the same time, bureaucratic rent-seeking behavior may create additional artificial shortages.

The WHO recommends that decision-makers in Member States conduct risk assessments through a mixed-methods approach to calculate the additional burden presented by possible importation of COVID-19 cases and establish policies on the basis of whether they have the capacity to manage this burden [44].

The mask collection plan adopted by Taiwan can secure the supply of masks in the hands of the government. More importantly, a risk-based approach should be adopted to prioritize meeting the mask needs of people with a high risk of infection. Those who need masks but have a low risk of infection should be encouraged to comply with hand hygiene, physical distancing, and other infection prevention and control measures, which are critical to prevent human-to-human transmission of COVID-19 [45].

## 5. Conclusions

The WHO has warned of shortages in face masks due to rising demand, panic buying, hoarding, and misuse [12]. The WHO has also recommended strategies to optimize the availability of personal protective equipment [19]. Kirubarajan et al. [20] revealed numerous strategies that have been evaluated for overcoming a limited supply of personal protective equipment during pandemics or epidemics. They grouped the strategies into six main categories: decontamination of disposable masks, reuse and/or extended wear of disposable masks, layering of masks, introduction of reusable respirators, use of non-traditional replacements or modifications to masks, and use of stockpiled or expired masks.

Allocating the use of medical resources through a reasonable economic system is also an important issue. However, there is a lack of discussion on the mechanism of allocating masks and plan evasion for the collection of personal protective equipment. The main contribution of this paper is the provision of a micro-foundation to analyze the mechanism of collection and plan evasion for face masks. Furthermore, we find that through the collection of masks and the Name-Based Mask Rationing System, the people’s right to procure masks can be guaranteed. However, due to asymmetric information, plan evasion by the NMT can occur. Because plan evasion can be caused by asymmetric information, sharing information with the public is also critical during a global pandemic.

In contrast to Taiwan’s collection of masks and the Name-Based Mask Rationing System, Wang et al. [46] suggested that the public wear masks during the COVID-19 pandemic according to the local context and that masks be distributed in accordance with the risk level. The WHO recommends that people wear face masks if they have respiratory symptoms or if they are caring for somebody with symptoms [47]. China’s national guideline has also adopted a risk-based approach in recommending the use of face masks among healthcare workers and the general public [48]. Feng et al. [49] pointed out that it is time to make rational recommendations on appropriate face mask use to complement recommendations on other preventive measures. It is critical to develop a dynamic system with flexible and agile operations that can quickly respond to evolving market conditions [50].

According to Tsang and Cheng [43], when an individual firm is a price taker, both the market and planned price influence its output. This could lead to the instability of output. In other words, fluctuations in the market and planned prices affect the profit-maximizing output of the individual firm. The supply curve thus has a kink, and plan evasion occurs regularly.

Expanding on the rent-seeking behavior described by Shleifer and Vishny [37] and the output models of NMTs by Zhou [38] and Huang and Lee [39], we discuss the NMT’s plan evasion under perfect and imperfect information. In this paper, assuming that the NMT is a price taker and that the planner is rational, we first derive the conditions under which a rational NMT will practice plan evasion when the planner has perfect information on the cost structure of the NMT. We then discuss the possibility of plan evasion when the planner has imperfect information on the NMT’s cost structure.

In summary, it is found that (i) if the rational planner has self-interest and perfect information, the NMT will not exercise plan evasion, and (ii) in the situation of imperfect information, with a low probability attributed to the high-cost firm (θ), less concern for the public interest (α), and a small cost ratio (the cost difference is significant), the planner has a tendency to impose a plan that makes the low-cost firm likely to fulfill it and the high-cost firm likely to exercise plan evasion.

In contrast to previous studies, this paper clearly introduces the role of the planner. When a planner has perfect information and is motivated by self-interest, the NMT will no longer practice plan evasion. As studies in the literature have pointed out, the government took action to control the supply and allocation of face masks, and this unique mask plan was believed to have greatly contributed to the successful prevention of COVID-19 transmission in Taiwan [4,5,6]. However, under imperfect information, plan evasion is quite probable due to the self-interest of the planner.

On 29 January 2021, the U.S. Centers for Disease Control and Prevention (CDC) issued a sweeping order requiring the use of face masks on nearly all forms of public transportation, as the country continued to report thousands of daily COVID-19 deaths [51]. Owing to the current shortage of masks, it is prudent to conserve them whenever possible. More than 100 billion masks will be needed for 300 million Americans annually if one person uses one mask per day. This is far beyond the current capacity of face mask manufacturers in the United States [52].

On the one hand, Taiwan’s experience shows that through the collection of masks and the Name-Based Mask Rationing System, the people’s right to procure masks can be guaranteed, and they have an equal probability of acquisition; however, plan evasion may be caused by plans with inappropriate quotas. Therefore, to promote market transaction efficiency, the government should adopt a lower quota for the collection of masks and allow manufacturers to freely sell them in the market after completing their plans. On the other hand, incorporating a risk-based approach into the collection of masks could be a more effective method to ensure that they are provided to people at a high or moderate risk of infection. Without effective public communication, a universal face mask wearing policy could result in societal panic and subsequently increase the nationwide and worldwide demand for face masks. These increased demands could cause a face mask shortage for healthcare workers and reduce the effectiveness of outbreak control in affected regions, eventually leading to a pandemic [53].

The limitations of this article are as follows: First, only plan evasion by firms on the NMT in a competitive market is discussed, but the actual market structure might be oligopolistic; second, the planner’s degree of concern for the public interest is set to be less than that of self-interest, but in fact, the planner might be more concerned about the public interest than self-interest. These model settings could lead to changes in the conclusions of this research.

Further study of the NMT’s plan evasion can be expanded by (i) using screening models to discuss the NMT’s plan evasion under imperfect information; (ii) including pricing power under an imperfect competitive market; and (iii) incorporating the demand uncertainty into this model in order to discuss the NMT’s plan evasion and planner’s optimum quota, among other factors.

## Figures and Tables

**Table 1:** Payoff matrix of the planner and the NMT^*^.

Planner *	NMT	
	The Type with High Cost (θ)	The Type with Low Cost (1−θ)
The planned quota and price in scenario (1): $q_{0}^{}=\frac{2P_{m}^{}}{3c_{h}^{}}$, $P_{0}^{}=\frac{P_{m}^{}}{3}$	$\frac{\left(8+\alpha \right)P_{m}^{2}}{18c_{h}^{}}$, $\frac{P_{m}^{2}}{18c_{h}^{}}$	$\left(\frac{4\left(1-\alpha \right)}{9c_{h}^{}}+\frac{\alpha }{2c_{l}^{}}\right)P_{m}^{2}$, $\left(\frac{1}{2c_{l}^{}}-\frac{4}{9c_{h}^{}}\right)P_{m}^{2}$
The planned quota and price in scenario (2): $q_{0}^{}=\frac{2P_{m}^{}}{3c_{l}^{}}$, $P_{0}^{}=\frac{P_{m}^{}}{3}$	$\frac{\left(2+\alpha \right)P_{m}^{2}}{18c_{h}^{}}$, $\frac{P_{m}^{2}}{18c_{h}^{}}$	$\frac{\left(8+\alpha \right)P_{m}^{2}}{18c_{l}^{}}$, $\frac{P_{m}^{2}}{18c_{l}^{}}$
The planned quota and price in scenario (3): $q_{0}^{}=\frac{P_{m}^{}}{c_{l}^{}\left(1-\alpha /2\right)}$,$P_{0}^{}=\frac{P_{m}^{}}{2\left(1-\alpha /2\right)}$	$\frac{\left(2-\alpha \right)P_{m}^{2}}{8c_{h}^{}{\left(1-\alpha /2\right)}_{}^{2}}$, $\frac{P_{m}^{2}}{8c_{h}^{}{\left(1-\alpha /2\right)}_{}^{2}}$	$\frac{\left(2-\alpha \right)P_{m}^{2}}{8c_{l}^{}{\left(1-\alpha /2\right)}_{}^{2}}$, $\frac{P_{m}^{2}}{8c_{l}^{}{\left(1-\alpha /2\right)}_{}^{2}}$

* Given the planned price and quota, the two entries in each box are the maximum expected return of the planner (left) and that of the NMT (right), respectively.

**Figure A1:**
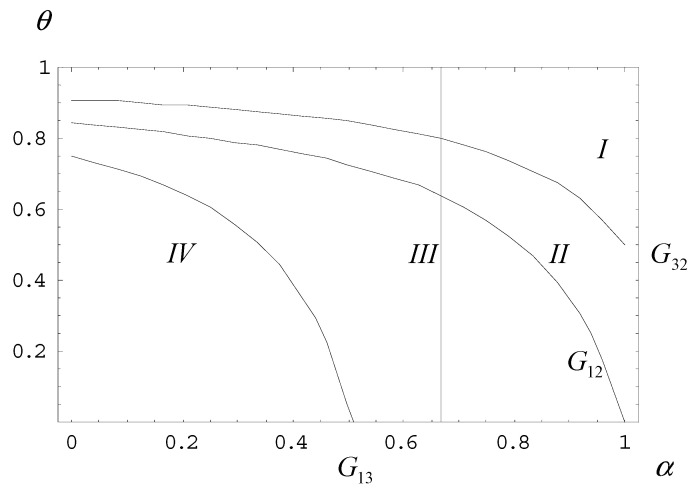
Decomposition of α, θ space for $c_{l}/c_{h}=0.2$.

## Data Availability

Not applicable.

## Appendix A. The Ranking of the Planner’s Expected Return among the Three Scenarios

In Figure A1, the three curves partition the (α,θ) space into four sections.

As mentioned above, curve $G_{12}$ traces the relation for $EG_{1}=EG_{2}$. Any point on the curve means that the expected return for scenario (1) ($EG_{1}$) equals that of scenario (2) ($EG_{2}$), and any point on the upper right of the curve indicates $EG_{1}>EG_{2}$. On the contrary, any point on the lower left of the curve means $EG_{1}<EG_{2}$. Curve $G_{13}$ mirrors $EG_{1}=EG_{3}$. Any point on the curve means that the expected return for scenario 1 ($EG_{1}$) equals that of scenario (3) ($EG_{3}$), and any point on the upper right of the curve means $EG_{1}>EG_{3}$. In contrast, any point on the lower left of the curve means $EG_{1}<EG_{3}$. The same logic holds for curve $G_{32}$, which traces $EG_{2}=EG_{3}$. Any point on the curve means that the expected return for scenario (2) ($EG_{2}$) equals that of scenario (3) ($EG_{3}$), and any point on the upper right of the curve implies $EG_{3}>EG_{2}$. On the other hand, any point on the lower left of the curve indicates $EG_{3}<EG_{2}$.

In Figure A1, Region I (to the right of $G_{32}$, $G_{13}$, and $G_{12}$) suggests that $EG_{3}>EG_{2}$, $EG_{1}>EG_{3}$, and $EG_{1}>EG_{2}$, respectively. That is, Region I in Figure 3 implies that $EG_{1}>EG_{3}>EG_{2}$. Given the parameter set in this area, the planner should design a plan in which both firms are expected to fulfill it in order to obtain the maximum expected return. Likewise, Region II implies $EG_{1}>EG_{2}>EG_{3}$. For a given set of parameters, in this area, the planner should design a plan in which both firms are expected to fulfill it in order to obtain the maximum expected return. Similarly, in Region III, we have $EG_{2}>EG_{1}>EG_{3}$. Within this framework, the planner should design a plan in which the high-cost firm exercises plan evasion, and the low-cost firm fulfills the target in order to capture the maximum expected return. Likewise, in Region IV, we have $EG_{2}>EG_{3}>EG_{1}$.

In Figure A1, Region I implies $EG_{1}>EG_{3}>EG_{2}$. Given the parameter set in this area, the planner should design a plan in which both firms are expected to fulfill it in order to obtain the maximum expected return. Likewise, Region II implies $EG_{1}>EG_{2}>EG_{3}$. Similarly, in Region III, we have $EG_{2}>EG_{1}>EG_{3}$. In Region IV, we have $EG_{2}>EG_{3}>EG_{1}$.

The decisions will be similar to those in Region III: Given the parameter set in this region, the planner should design a plan in which the high-cost firm practices plan evasion, while the low-cost firm fulfills the plan in order to obtain the maximum expected return. Furthermore, if the point is to the right of curve $G_{12}$ (including Region II, Region I, and curve $G_{32}$), the planner will choose a plan in which scenario (1) takes place. In contrast, if the point is to the left of curve $G_{12}$ (including Region II, Region III, and curve $G_{13}$), the planner will choose a plan in which scenario (2) will occur. The planner will not adopt any plan in which scenario (3) prevails.
